# The surviving sepsis campaign: basic/translational science research priorities

**DOI:** 10.1186/s40635-020-00312-4

**Published:** 2020-07-17

**Authors:** Clifford S. Deutschman, Judith Hellman, Ricard Ferrer Roca, Daniel De Backer, Craig M. Coopersmith, for the Research Committee of the Surviving Sepsis Campaign, Craig M. Coopersmith, Daniel De Backer, Massimo Antonelli, Clifford S. Deutschman, Laura Evans, Ricard Ferrer-Roca, Judith Hellman, Sameer Jog, Jozef Kesecioglu, Ishaq Lat, Mitchell M. Levy, Flavia Machado, Greg Martin, Ignacio Martin-Loeches, Mark E. Nunnally, Andrew Rhodes

**Affiliations:** 1 Department of Pediatrics, Hofstra/Northwell School of Medicine and the Feinstein Institute for Medical Research/Elmezzi Graduate School of Molecular Medicine, Manhasset, NY USA; 2 Department of Molecular Medicine, Hofstra/Northwell School of Medicine and the Feinstein Institute for Medical Research/Elmezzi Graduate School of Molecular Medicine, Manhasset, NY USA; 3 Department of Anesthesia and Perioperative Care, University of California, San Francisco, San Francisco, CA USA; 4 Intensive Care Department, Vall d’Hebron University Hospital, Barcelona, Spain; 5 Shock, Organ Dysfunction and Resuscitation (SODIR) Research Group, Vall d’Hebron Institut de Recerca, Barcelona, Spain; 6 Chirec Hospitals, Université Libre de Bruxelles, Brussels, Belgium; 7 Department of Surgery and Emory Critical Care Center, Emory University, Atlanta, GA USA

**Keywords:** Basic science, Research, Sepsis, Surviving sepsis campaign, Translational science

## Abstract

**Objectives:**

Expound upon priorities for basic/translational science identified in a recent paper by a group of experts assigned by the Society of Critical Care Medicine and the European Society of Intensive Care Medicine.

**Data sources:**

Original paper, search of the literature.

**Study selection:**

This study is selected by several members of the original task force with specific expertise in basic/translational science. Data extraction and data synthesis are not available.

**Conclusions:**

In the first of a series of follow-up reports to the original paper, several members of the original task force with specific expertise provided a more in-depth analysis of the five identified priorities directly related to basic/translational science. This analysis expounds on what is known about the question and what was identified as priorities for ongoing research. It is hoped that this analysis will aid the development of future research initiatives.

The Sepsis-3 Task Force, jointly sponsored by the Society of Critical Care Medicine (SCCM) and The European Society of Intensive Care Medicine (ESICM), recently redefined sepsis as “life-threatening organ dysfunction caused by a dysregulated host response to infection” [1]. One objective underlying this new definition was to refocus basic research into the pathobiology of this deadly syndrome. Specifically, the Task Force members sought to (1) emphasize that organ dysfunction is a key, defining characteristic of sepsis; (2) broaden the scope of investigation to include aspects of the host response beyond immunologic changes; and (3) direct studies toward identifying the links between the host response to infection and the development of organ dysfunction. The papers that resulted from the work of the Task Force have generated both enthusiasm and controversy. Most of the response, however, has focused not on the redefinition but rather on the evidence-based clinical criteria used by the Task Force to identify patients with sepsis from patients with uncomplicated infection. The definition is inherently difficult to examine in the clinical arena because clinical identifiers of “organ dysfunction” and “dysregulated host response” are limited. Therefore, the Task Force explicitly stressed that future research into the basic science of sepsis focus on clinically applicable identifiers of “organ dysfunction” and “dysregulated host response.”

A second major collaboration between the SCCM and the ESICM is the Surviving Sepsis Campaign (SSC). In formalizing their joint responsibilities for the SSC, the leaders of both societies established a Research Committee. The first task of the committee was to identify future research priorities. The deliberations of the committee led to the joint publication of “Surviving Sepsis Campaign Research Priorities for Sepsis and Septic Shock” in the journals *Critical Care Medicine* and *Intensive Care Medicine* in August 2018 [2, 3]. The initial document presented a broad overview of research priorities in several critical care domains with an expressed intention to, in the future, publish separate papers with more detailed descriptions for each domain. This paper, focusing on basic/translational science, contains the effort to fulfill that promise.

## Methods

The content of the initial paper published by the SSC Research Committee [2, 3] was developed by asking each committee member to identify the research questions that most urgently required answers. Using a modified Delphi approach, the Task Force members reduced the original 88 suggestions to a series of questions focused on clinical care and four directed toward “basic science.” The final queries were presented in the original publication. Three Task Force members with specific expertise were tasked with generating expanded reviews of the four basic science questions as well as one additional query focusing on epigenetics. Each question was addressed by one of the three task force members. These in-depth reviews were then edited by the group as a whole, with added input from the committee co-chairs. The final result is presented here.

## Overview of the presentation

The five basic science questions identified by the Task Force as a whole are as follows:

1. What mechanisms underlie sepsis-induced cellular and subcellular dysfunction?
2. How does sepsis alter bioenergetics and/or metabolism (both enhancement and failure)?
3. How does sepsis (and/or approaches used to manage sepsis) alter phenotypes and interactions in the host microbiome and do alterations in the microbiome affect outcomes?
4. How do epigenetics influence the pathobiology of sepsis?
5. What mechanisms initiate, sustain, and terminate recovery after sepsis?

The format for each of the five questions directly mirrors that used in the previously published overview, which contains a more extensive description of the methods [2, 3].

### Question 1: What mechanisms underlie sepsis-induced cellular and subcellular dysfunction?

#### What is known

The redefinition of sepsis by the Sepsis-3 Task Force focuses on the importance of organ dysfunction as the sine qua non of the disorder [1]. As a result, there is a compelling need to develop new clinical constructs for dysfunction in individual organ systems, as well as to continue to advance animal models to more accurately reflect human sepsis [4]. The process is hampered by (1) lack of a true gold standard to identify sepsis and (2) a need to develop indices of organ dysfunction that can be measured in patients, where access to cells is limited. There is little to be done about the first point. To address the second, it will first be necessary to identify the cellular abnormalities that underlie dysfunction in specific organ systems. These abnormalities can then be related to updated proxy measures that correlate, as closely and specifically as possible, with the actual cellular changes that lead to sepsis-induced organ dysfunction. However, access to clinical samples is limited, most often to what can be obtained from sampling blood, urine, or less frequently with biopsy or lavage specimens. Thus, WBCs are likely to be all that is routinely available. As a result, there is an imperative to use either models or in silico constructs to identify cellular abnormalities consistent with sepsis-induced dysfunction in different organs. Correlation of these abnormalities with measurements that can be made using blood or urine, or with noninvasive functional testing (e.g., echocardiography) will provide a clinically useful approach to organ dysfunction.

A) Pathobiological changes that constitute “dysfunction” have been identified in several organ systems. These abnormalities reflect a small number of identifiable patterns.

1. Some pathobiological abnormalities are consistent with changes in clinical variables that are commonly measured. These changes can therefore be used to indicate the presence of organ dysfunction and to follow its clinical course. For example, pathologic abnormalities in the lung (edema in intra-alveolar septa and air spaces, debris—the “hyaline membrane”—lining alveoli, loss of type 1 pulmonary epithelial cells via necrosis and apoptosis, loss of synthetic capacity by type-2 cells) [5–7] are consistent with the clinical presentation of lung injury (development of hypoxemia, reduced compliance, atelectasis, and multifocal lung consolidation) that are often used to assess respiratory function in patients.
2. In other cases, changes consistent with organ dysfunction have been identified under experimental conditions and in animal models. However, these variables are not routinely measured clinically, nor have surrogates that correlate with these abnormalities been clinically validated. For example, sepsis-induced abnormalities in hepatic detoxification (e.g., transcellular bile acid transport) and biosynthesis (e.g., hepatocellular gene expression) have been described at the basic level [8–10]. However, these variables are not currently measured in the clinical realm, where hepatic dysfunction from any cause is assessed using serum levels of transaminases and elevations of bilirubin that are relatively nonspecific and not diagnostic of sepsis-induced hepatocellular dysfunction. Thus, a better understanding of pathobiology is required to identify measurements with clinical utility.
3. Although some aspects of sepsis pathobiology have been well-described, their significance can be interpreted in two completely contradictory ways. As an example, consider sepsis-induced abnormalities in WBCs, which include elevated cytokine elaboration, reduced bacterial killing/clearance, and depressed responses to stimulation with inflammatory agonists [11]. However, elevated cytokine expression can reflect the response to two different, diametrically opposite stimuli. High levels of tumor necrosis factor (TNF)-α or interleukin (IL)-6 can induce inflammation and may directly damage cells. Thus, the increased elaboration might in itself be pathologic; appropriate therapy would therefore be blockade of their activity. However, in animal models of sepsis, there is impairment of intracellular pathways that are activated by these cytokines—that is, cells do not have the expected response to cytokines. The adaptive response to this situation would be to overcome the defect with increased cytokine elaboration and release, much as primary adrenal insufficiency results in enhanced release of thyroid-stimulating hormone from the pituitary [12]. If elevated levels of TNF-α and IL-6 represent a compensatory mechanism to counteract these defects, therapy would involve increasing TNF/IL-6 abundance or activity even further. It is conceivable that both possibilities can occur concurrently. That is, the response of some cells to cytokines may be inadequate (e.g., hepatocytes, as identified in animal models), which drives up levels of IL-6 and TNF-α. These high levels, however, may be toxic to other cells (e.g., pulmonary or gastrointestinal tract [GI] epithelial cells). In effect, the elaboration and secretion of cytokines may be driver of pathology, a beneficial response to pathology, both, or an incidental change. Thus, while it is clear that sepsis elevates TNF-α/IL-6 levels, a better understanding of the impetus underlying these changes is required to intervene effectively.
4. In some cases, it may be impossible to separate sepsis pathobiology from the effects of treatment. The best example lies in attempts to differentiate acute respiratory distress syndrome (ARDS) from ventilator- or fluid-induced lung injury. A better understanding of differences in the cellular pathobiology of these disorders is therefore required.
5. Certain aspects of sepsis pathobiology have proven remarkably difficult to understand. Evidence may suggest the presence of global organ dysfunction and of abnormalities in processes specific to certain cell types, but it is difficult to sort out the complex combination of dysfunction in individual types of cells and the interaction between these cells. Consider sepsis-induced acute kidney injury. Identified specific abnormalities include (1) reduced glomerular filtration rate (GFR) despite, (2) adequate or high renal blood flow (RBF) [13], (3) poorly understood defects in tubular function [14], and (4) aberrant or attenuated responses to exogenous modulation, for example, by hormones. Although a comprehensive explanation that encompasses all of these defects has been proposed [14] and appears to be present in a rodent model [15], it has not been validated in large animals or in clinical sepsis. Decreased GFR despite adequate or supra-normal RBF can reflect dilation of the efferent (postglomerular) arteriole, but how this change might relate to altered resorption and transport in the tubules is unknown. Correlations between changes in biomarkers for GFR (serum levels of creatinine, neutrophil gelatinase-associated lipocalin [NGAL], or cystatin-C) and those reflecting tubular dysfunction (kidney injury marker [KIM]-1, insulin-like growth factor binding protein [IGFBP]-7) are lacking. How KIM-1 and NGAL IGFBP-7 are related to actual defects in tubular function is unclear. Further, it is difficult to differentiate the risk of renal compromise (often termed “stress”) from actual dysfunction or injury. That is, an early period of inflammatory activation and perhaps proximal tubular dysfunction/injury precedes abnormalities (uremia, acidosis, abnormal electrolyte levels such as hyperkalemia) that have historically been used to indicate a need for renal replacement therapy. As is the case with other organs (e.g., heart, liver), the correlation between dysfunction and histologic evidence of damage is obscure. Thus, a far more complete understanding of the pathobiologic changes that cause dysfunction in different types of cells and/or processes in individual organ systems and of how sepsis disrupts interactions between different types of cells is of key importance.
  B) Sepsis pathobiology may reflect dysfunction is closely related cell types that are present in a number of distinct organ systems. For example, experimental and clinical evidence indicates the presence of sepsis-induced defects in protein synthesis in hepatocytes, type 2 pulmonary epithelial cells, and “central,” that is, pituitary, endocrine cells [5–10, 16–20]. Conversely, each cell or type of cell may develop a specific defect or manifest dysfunction in a unique manner. For example, sepsis upregulates production and release of cytokines by monocytes and lymphocytes [11] but decreases production and release of surfactant or surfactant proteins by type II pulmonary epithelial cells [5–7] or of hormones by endocrine or pituitary cells [18–20].
  C) As mentioned above, sepsis is associated with defects in cytokine signal transduction pathways. Data suggest that this type of abnormality may alter the response to other mediators. Impairment of endocrine signal transduction in sepsis has been well-described [19]. Sepsis also downregulates β-adrenergic and other G-protein receptor-mediated pathways [21, 22], which contributes to sepsis-induced cardiac dysfunction [23]. More recent data indicate defective signal transduction in pathways responding to steroid hormones, for example, glucocorticoids [24].
  D) There is an important extension of the described defects in cytokine- and hormone-mediated cellular responses. Cytokines, which are primarily produced by immune and endothelial cells, and hormones, which circulate, represent pathways by which cells in one tissue or organ system can be “informed” of changes that occur elsewhere. For example, a decrease in blood pressure or oxygen content in the blood supplying the brain is sensed by specialized cells of the carotid artery and trigger the release of epinephrine, vasopressin, and angiotensin, which in turn increase cardiovascular function, thereby restoring substrate delivery to the brain. Similarly, immune and endothelial cells respond to the presence of either damage-associated molecular patterns (DAMPs) or pathogen-associated molecular patterns (PAMPs) by elaborating and releasing cytokines, which can then alter activity in adjacent cells. Activation of circulating immune cells makes it possible for the transmission of DAMP/PAMP—induced responses to remote cells, tissues, and organ systems. In effect, endocrine and immune changes function, in part, as part of an organism-wide communications system. Sepsis impairs these forms of communication by attenuating the ability of cells to respond to cytokine and endocrine mediators.

A third major long-range communication system within organisms is the nervous system. Multiple lines of evidence indicate that sepsis induces impairment in both the peripheral nervous system and CNS [25–28]. The peripheral neuropathy of sepsis/critical illness has been well-described. Several more recent findings have demonstrated that sepsis impairs signaling originating in the brain that limits responses in other organ systems. The orexinergic system of the hypothalamus was recently reported to modulate, at least in part, depressed activity/arousal, bradycardia, hypothermia, and hypopnea in experimental sepsis (analogous to the tachycardia, hyperthermia, and tachypnea that initially characterize the human response). This defect in orexinergic signaling also alters pituitary hormone release. All these changes were reversed when orexin was administered into the cerebrospinal fluid [27]. Finally, sepsis interferes with the “inflammatory reflex,” a negative feedback loop in the vagus nerve where ascending signals “inform” the brain of inflammatory events in the periphery while descending signals limit the cytokine response to those same responses [28–31]. A number of studies have demonstrated brain inflammation in experimental sepsis, most often implicating microglia [29, 30].

E) A substantial body of evidence suggests that sepsis causes a global defect in a basic cellular or subcellular function in many cell types. The ubiquitous presence of such an abnormality would produce dysfunction in many different cell types, irrespective of their specific function or location. For example, there are numerous reports of sepsis-induced mitochondrial dysfunction in multiple cell types [19, 20, 27]. Abnormalities have been reported in mitochondrial oxidative phosphorylation, with impairment described in all four complexes of the electron transport chain (ETC) [32–36]. The resulting energy deficit could disable cell-specific functions in any cell that is mitochondria-dependent.
F) Finally, dysfunction in a single type of cell that is present in virtually all organs could underlie cell- and organ-specific dysfunction. For example, endothelial cells, which are present in all tissues, actively produce inflammatory mediators and coagulation intermediaries during sepsis and contribute to sepsis-induced vascular dysfunction and leak [18, 37]. Thus, differential or sequential development of endothelial dysfunction in different vascular beds might mediate aspects of sepsis-induced organ dysfunction.

#### What is not known—gaps in our understanding—directions for future research

1. Does a global defect that is shared by multiple cell types underlie all forms of sepsis-induced cellular dysfunction?
2. Are there unique mechanisms of dysfunction that are specific to different types of cells, including different types of cells within a single organ?
3. Do cells of similar embryologic origin (e.g., epithelium) become dysfunctional in ways that differ from other types of cells?
4. Do cells with similar functions (e.g., elaboration/release of proteins, lipids) develop unique forms of dysfunction that differ from that of cells with different basic functions (e.g., all cells that contract)?
5. Endothelial cells are present in virtually all organ systems and may directly modulate organ function. Does endothelial cell dysfunction underlie dysfunction in other organ systems?

### Question 2: how does sepsis alter bioenergetics and/or metabolism (both enhancement and failure)?

#### What is known

Many of the effects of sepsis on bioenergetics and/or metabolism have been well-described [38, 39]. In general, sepsis is associated with an increase in metabolic rate, as reflected in oxygen consumption and overall substrate utilization [40]. There is, however, a reduction in adenosine triphosphate (ATP) utilization in many tissues [41–44]. This limitation occurs in concert with maintenance of ATP abundance, suggesting that the decreased use reflects an attempt to conserve ATP availability to avoid cell death [45]. In this setting, “aerobic glycolysis” is stimulated and assumes a greater role in ATP production. That is, despite what would appear to be more than adequate oxygen availability, substrate is shunted into the glycolytic pathway, resulting in the generation of lactate. Sepsis differs from other states where aerobic glycolysis is present (e.g., aerobic exercise) because of the ability of the liver to use lactate for gluconeogenesis (the Cori cycle) or bicarbonate is impaired [46]. This limitation contributes to hyperlactatemia and a metabolic acidosis. Thus, the aerobic glycolysis of sepsis constitutes a clinical example of the Warburg effect [47–49].

Two explanations for the sepsis-induced changes in glycolysis have been advanced. The first posits that increased glycolysis in sepsis reflects a defect in the microvasculature that impairs the delivery of oxygen to metabolically active tissues. Interestingly, the changes may be attenuated by hemodynamic targeted interventions [50]. The alternative implicates a defect in mitochondrial function that leads to a decrease in oxygen utilization. The latter is supported by abundant evidence of defective oxidative phosphorylation in sepsis. Importantly, these two putative mechanisms are not mutually exclusive; indeed, evidence suggests that both are operative in sepsis. Which specific complexes in the ETC are impaired is incompletely understood, and appears to depend on the tissue, or, in animals, the model. Decreased activity has been identified in each of the five complexes in ETC [33, 34]. Importantly, complex II serves as an enzyme in both oxidative phosphorylation and in the Krebs cycle. Therefore, impairment would enhance the diversion of substrate into glycolysis, a change that has been hypothesized to underlie the Warburg effect [51]. One of the consequences of impaired mitochondrial function, whether as a result of a defect in either the microcirculation or the mitochondria themselves, has been termed “hibernation” or “oxygen conformance” [52–54]. In this state, nonvital functions are shut down in order to maintain cell viability despite inadequate oxygen delivery or utilization.

Sepsis is also known to alter substrate preference, with a relative decrease in the utilization of glucose (glucose intolerance) relative to fat and protein [55–57]. As a result, septic patients tend to be hyperglycemic. In later stages, oxidation of fatty acids may also be impaired, as reflected in elevated serum levels of lipoproteins, free fatty acids, and triglycerides [47–49]. There is accelerated catabolism of skeletal muscle and possibly smooth muscle as well [58]. In addition, the effects of micronutrient (e.g., vitamins, trace metals) are also impaired, reflecting either deficiency or altered activity [59].

Changes in metabolism may also reflect the influence of “communications” pathways (described under question 1)—humoral (i.e., cytokine/white cell-mediated), endocrine, neuronal—on cellular function. Cytokine-mediated changes are well-described; the initial description of the metabolic effects of TNF was based on observations in diseased cattle—cachexia despite grossly lipemic serum. Studies in clinical sepsis and/or animal models have identified impaired activity of hormones that are known to affect metabolism (e.g., insulin, glucagon, T3/4, growth hormone, epinephrine/norepinephrine cortisol) and of less well-studied endocrine agents (adiponectin, leptin, ghrelin). Indeed, relative endocrine resistance is a characteristic finding [60]. Some studies suggest that sepsis alters the CNS effects of leptin and ghrelin, which are integral in central modulation of metabolism [61, 62]. Recent studies implicate alterations in the activity of the orexinergic and basal forebrain muscarinic systems in the brain in sepsis-induced metabolic changes [27, 28].

Several recent cohort studies and meta-analyses have suggested a protective role for obesity in critically ill patients [63–65]. However, the data are far from conclusive and mechanistic explanations are lacking. Indeed, basic studies in mice suggest that diet-induced obesity increases sepsis-induced inflammation as well as injury to the heart and liver [66–69].

#### What is not known—gaps in our understanding—directions for future research

1. What causes the increased metabolic rate noted in sepsis?
2. What mechanisms mediate alterations in oxidative phosphorylation? In particular, what underlies the altered activity in specific ETC complexes?
3. What mechanisms alter sepsis-induced changes in pathway (e.g., glycolysis, beta-oxidation, nitrogen cycle), substrate (e.g., carbohydrate, fat, protein, micronutrient), and/or cell (e.g., cardiomyocyte, hepatocyte) specific metabolism?
4. What mechanisms underlie sepsis-induced defects in endocrine activity?
5. How does sepsis affect brain circuits that control metabolism?
6. How do cytokines alter metabolic pathways?
7. Do metabolic pathways influence inflammation, and if so, how?
8. Are changes in energetics observed in all cells or are they cell-type-specific?
9. Are defects affecting energetics present only in mitochondria or are there also changes in other subcellular structures?
10. Is obesity protective against sepsis? Why are results in human sepsis and animal models discrepant?

### Question 3: how do the microbiota and the microbiome contribute to the pathobiology of sepsis?

#### What is known

In a number of disease states, pathology is determined by an alteration in the interactions between the host and its complex microbial ecosystem. These changes, which likely affect outcomes of critically ill and septic patients, are the subjects of intense clinical and basic science investigations. The development of culture-independent methods to detect microbial genes has revolutionized this research and greatly enhanced microbe identification [70–72]. We now appreciate the enormous diversity of microbial species (microbiota) and microbial genomes (microbiome) that exist within human ecosystems (e.g., GI tract [gut], lungs, skin, etc.), as well as the importance of the microbiome to human health and disease [70]. It has become clear that the gut microbiome plays an important role in patients with sepsis, suggesting that the microbiome and host-microbiome interactions may be therapeutic targets [73].

The average healthy 70 kg adult human male is estimated to contain approximately 30 trillion host cells and to be colonized by nearly 40 trillion microbes [72, 74]. The gut microbiome is the largest in the human body and, perhaps because it can be assessed using fecal samples, has been the most extensively studied microbiome. The gut contains over 1000 species of bacteria, fungi, protozoa, and viruses containing nearly 9.9 million microbial genes [75]. These microbes live in complex and interactive communities and exhibit extensive geographic heterogeneity [75].

Recent studies indicate that the gut microbiome contributes to the regulation and maturation of biological function both within and outside of the GI tract [76]. The gut microbiome contributes to (1) pathogen containment, (2) immune maturation and functionality within and outside of the GI tract, (3) neurologic signaling, (4) host cell proliferation, (5) toxin elimination and drug modification, and (6) biosynthesis of compounds, including vitamins and neurotransmitters [77–84]. Dysbiosis, the development of imbalances in the composition and/or function of the host microbiome, has been implicated in a wide variety of human and animal disorders, including cardiovascular disease, autism spectrum disorders, metabolic disorders, and asthma [85–91]. Importantly, host-microbiota interactions are bi-directional; the two impact each other enormously.

A number of studies have suggested that the host microbiome is altered both by sepsis itself and by management approaches used to treat septic patients. Some have gone so far as to postulate that these changes contribute to the multiple organ dysfunction syndrome (MODS) [76, 92–94]. Alverdy and Krezalek [95] defined mechanisms by which they believe the microbiome becomes a “pathobiome” [95–99] that leads to “nonresolving MODS” [95, 96, 100].

A) Multiple endogenous host and external factors contribute to the conversion of a healthy microbiome into a pathobiome in patients with sepsis.

Clinical studies have documented changes in the number of microbial species and genes, pathogenicity of different microbes, and microbial production of metabolites in the gut microbiome of adult patients following hepatectomy, trauma, cardiac arrest, and cerebrovascular events [101–104]. Clinical studies in the ICU arena have primarily focused on the microbiome of the general ICU population and not specifically on the subset of patients with sepsis. Sepsis-induced changes were suggested by a small clinical study of patients with severe systemic inflammatory response syndrome caused by sepsis (*n* = 18), trauma (*n* = 6), and burns (*n* = 1) [102] and likely contribute to findings in critically ill adults [100] and children [104]. Enrichment of the lung microbiome with gut bacteria has been reported in mice that have undergone cecal ligation and puncture (CLP) [105], a widely used model of abdominal sepsis that was first described in 1980 [106], and in critically ill patients with ARDS [105]. Finally, alterations in the gut microbiome have been reported in mice following experimental brain injury [107].

A number of factors alter the composition and pathogenicity of the human microbiome (Table 1) [73, 75, 78, 108–114]. Although the relative contribution of each of these factors to clinical changes in the microbiome during critical illness is difficult to discern, antibiotics and severity of illness seem to be major determinants [73].

**Table 1:** Factors contributing to the development of gut dysbiosis

	Factors	References
Exogenous	Geographic location	[75]
	Exposures to microbes in the environment	[110]
	Diet	[112]
	Drugs: antimicrobial agents, immunosuppressive agents, etc.	[78, 108]
Host factors	Genetic factors	[109, 111]
	Immune health/responses	[115]
	Diseases/disorders/infections	[76, 109]
	Severity of critical illness	[73]

Basic research directed at identifying the mechanisms underlying both gut dysbiosis and changes in the microbial virulence in species such as Candida, Staphylococcus, and Pseudomonas [79, 97, 99] has identified a number of contributors. Factors involved in the latter are both intrinsic (hormones, endorphins) and extrinsic factors (opiates, antibiotics, immunosuppressive agents). Recent studies have invoked quorum sensing, a complicated system that allows microbes to collectively respond to the environment [97, 116, 117]. Pseudomonas aeruginosa has been reported to adopt a more virulent phenotype in response to exogenous and endogenous opioids via quorum sensing mechanisms [97].

B) Gut dysbiosis and the development of a pathobiome is associated with worse outcomes in septic patients.

Clinical studies suggest that sepsis is associated with the replacement of a diverse, health-promoting microbiome by a pathobiome that is harmful to the host [92, 94, 118, 119]. Animal studies support the contribution of this dysbiosis to septic pathobiology and MODS [120–122]. Dysbiosis is characterized by an altered microbiome that eliminates microbes such as Lactobacillus and Bifidobacterium that protect against overgrowth and limit epithelial adherence of pathogenic bacteria [123, 124]. The protective effects of the healthy microbiome may lie in the ability of Lactobacillus and obligate anaerobes to ferment nondigestible dietary fibers to short-chain fatty acids (SCFAs), including propionate, acetate, and butyrate, that can be used by the gut epithelium to promote barrier integrity and enhance immune function [124–126]. This loss can promote systemic infection and dysregulated inflammation.

C) Manipulation of the gut microbiome affects outcomes from infection and alters immune responses in some conditions.

Therapeutic manipulation of the gut microbiome via selective decontamination, fecal transplantation, or use of probiotics has been attempted in several conditions attributed to gut dysbiosis. This approach is supported by studies in animals [127–133]. A number of studies have documented that selective gut decontamination in both humans and animals reduced organ inflammation and dysfunction [134, 135]. Fecal transplantation has shown efficacy for severe recurrent diarrhea caused by antibiotic-resistant *Clostridium difficile* and has mixed results in inflammatory bowel disease, but its utility in critically ill patients has yet to be defined [136–139].

D) What environmental factors could be manipulated to promote a healthy microbiome?

Although it may be difficult to forgo treatment with essential therapies such as antibiotics, other factors, such as diet, stress ulcer prophylaxis, and the use of immunosuppressive agents, are potentially amenable to interventions. Although some cannot be changed (e.g., host genome), understanding their impact on the host’s microbiome may eventually lead to personalized approaches to manipulating the host’s environment to promote a healthy microbiome.

E) How do the microbiomes from different parts of the body determine outcomes?

Thus far, studies on how the changes in the microbiome affect critically ill patients have focused on the gut. However, critical illness and sepsis also cause changes in other microbiomes, such as the lung [105]. The importance of non-GI microbiomes in determining outcomes of sepsis has yet to be defined.

#### What is not known—gaps in our understanding—directions for future research

1. How do specific approaches to the management of sepsis affect the host microbiome?
2. Which of the factors noted in Table 1 promote gut dysbiosis? What are their specific effects? Are other factors are involved?
3. Do changes in the microbiome directly lead to the development or exacerbation of sepsis/MODS? What specific components of the pathobiome are of particular interest?
4. Does the conversion from a normal microbiome to a pathobiome cause/exacerbate sepsis MODS or is the change merely associated with sepsis/MODS?
5. Does manipulation of the microbiome alter the incidence, severity, or outcomes of sepsis?
6. Can the pathobiome be stably converted back to a normal microbiome? Is such a conversion beneficial?
7. Does the presence/absence of SCFA affect the pathogenesis of sepsis? Does it affect organ dysfunction or sepsis outcomes? Does administration of SCFAs or the introduction of SCFA-producing bacteria alter sepsis pathogenesis and/or improve organ dysfunction and outcomes?
8. What environmental factors could be manipulated to promote a healthy microbiome? Does manipulating diet, exercise, or immunosuppressive agents affect the development of sepsis/MODS? Does it alter outcomes?
9. Can a better understanding of how host genomics interact with specific elements in the microbiome/pathobiome provide insight that can alter outcomes? Can this understanding be leveraged to provide personalized approaches to the promotion of a healthy microbiome?
10. Are prebiotics or probiotics therapeutically beneficial? Is fecal transplantation beneficial?

### Question 4: how do epigenetics influence the pathobiology of sepsis?

#### What is known

“Epigenetics” is a catch-all phrase used to describe alterations in gene expression that occur independent of changes in DNA sequence. It is increasingly evident that these alterations are essential determinants of human health and diseases [140]. Epigenetic alterations are dynamic, and under optimal conditions, serve beneficial functions. However, these changes can also contribute to the development of major diseases and disorders, as well as affecting outcomes in more subtle ways. Importantly, epigenetic changes can be heritable. Epigenetic changes may alter the host response to infection and injury, and therefore the ability to prevent or clear infection.

Epigenetic mechanisms include DNA methylation, histone modifications (methylation, acetylation, phosphorylation, ubiquitination, and glycation), and elaboration of microRNAs (miRNAs). All can lead to rapid, transient, and reversible modification of gene expression by inducing gene activation or silencing. Studies in humans and animals suggest that epigenetic modifications impact sepsis [140, 141].

A) Sepsis and endotoxemia are associated with epigenetic alterations in myeloid cells and in modulation of inflammation.

DNA methylation, histone modifications, and altered levels of miRNAs have been observed in cells in endotoxemia and sepsis. The changes in gene expression associated with these modifications modulate responses that may lead to endotoxin tolerance, immunosuppression, and immunoparalysis and susceptibility to infections [142–152]. DNA and histone methylation can silence genes encoding inflammatory mediators, anti-oxidants, and other factors that contribute to systemic inflammation [146, 152, 153]. In contrast, DNA methylation at certain genes has been linked to excessive systemic inflammation [147, 149].

B) Lung injury induces epigenetic modifications that promote vascular permeability.

Experimental lung injury induced modification of histone H3 and thus downregulation of angiopoietin 1, Tie2, and Vegfr2. These changes, in turn, have been implicated in the expression of genes encoding inflammatory mediators and in the development of excessive microvascular permeability [154].

C) Histones and miRNAs circulate in sepsis and may contribute to organ dysfunction.

Animal and human studies have shown that histones circulate in sepsis and are associated with increased mortality rates [155, 156]. miRNAs have also been found to circulate in human sepsis [157–160]. In CLP mice, miRNAs that circulate in extracellular vesicles have been implicated in the development of inflammation [161].

D) Epigenetic alterations are being explored as potential sepsis biomarkers and therapeutic targets.

Epigenetic alterations, including DNA methylation, histone modifications, and miRNAs, have been touted as diagnostic biomarkers, markers of disease severity, and therapeutic targets [155, 162–164]. Studies on utility, which has been demonstrated in cancers [165], are being explored in sepsis [163, 166–172].

#### What is not known—gaps in our understanding—directions for future research

1. Are epigenetic changes such as DNA methylation, histone modifications, and miRNA expression important contributors to the development of sepsis?
2. Do epigenetic changes contribute to sepsis-induced MODS or changes in immune function? Or are they merely epiphenomena?
3. Can epigenetic changes be used as diagnostic and/or prognostic biomarkers?
4. Are epigenetic alterations potential therapeutic targets in sepsis?
5. Might existing drugs (e.g., HDAC modulators) be used or new drugs be designed to target epigenetic alterations in sepsis?
6. How do epigenetic factors influence the microbiome, and vice versa, and what are the implications for sepsis?

### Question 5: what mechanisms initiate, sustain, and terminate recovery after sepsis?

The phrase “recovery from sepsis” can have a broad meaning. On an organism, wide level recovery can mean survival, reversal of organ dysfunction, or resumption of premorbid activities. Control/elimination of the focus of infection is required but it is by no means sufficient. Indeed, organ dysfunction can persist for a prolonged period afterward; some sepsis-induced abnormalities may never fully resolve. Overall, recovery likely reflects reversal of a vast array of maladaptive cellular, subcellular, and biochemical changes that develop during sepsis. These alterations involve metabolism, dysfunction in organelles, in particular mitochondria, attenuation of intracellular signal transduction pathways, and decrement in the activity of WBCs, neuronal pathways, and endocrine responses that are responsible for the transfer information from one part of the organism to another.

Despite reports that describe the events occurring during recovery or lack of recovery, the specific mechanisms that initiate recovery are unknown. It is possible that recovery represents an extension of Darwinian theory: sepsis resolves and the fittest cells/patients survive by implementing general mechanisms of protection. Alternatively, it may be that specific molecules/processes/events directly promote recovery from sepsis. Or perhaps recovery represents a combination of both.

A) What immunological processes contribute to recovery from sepsis?

In contrast to a substantial body of work describing the resolution of “normal” or “balanced” inflammation, much about the reversal of the unique inflammatory state present in sepsis is poorly understood. Perhaps the greatest hindrance lies in a limited ability to characterize that state. To some extent, sepsis resembles an exaggeration of normal inflammation, with expression of high levels of pro-inflammatory cytokines. Other aspects, such as white cell bacterial engulfment and bacterial killing, are impaired. However, the combination of immune excess and immure incompetence characteristic of sepsis is strikingly unusual. It is logical to assume that the resolution of sepsis will be similarly difficult to understand. Thus, the complexity of the issue is apparent and requires attention.

1. Do anti-inflammatory cytokines contribute to recovery after sepsis? If so, how?

#### What is known

Resolution of sepsis-induced inflammation is an active process that likely involves the production of endogenous anti-inflammatory molecules. A number of anti-inflammatory cytokines are upregulated during the course of sepsis (e.g., IL-1RA, IL-4, IL-10, and TGF-β) [173–175]. Importantly, IL-4 alters the balance between pro-inflammatory and anti-inflammatory T lymphocyte phenotypes and participates in the regulation of proliferation, differentiation, and apoptosis in multiple cell types. These IL-4-mediated changes are important for resolution of nonseptic inflammation but may actually contribute to the pathobiology of sepsis [176]. IL-10, in turn, blocks synthesis of interferon-γ, IL-1, TNF-α, IL-12, and GM-CSF and enhances the endocytosis-mediated elimination of human leukocyte antigen (HLA)-DR for the surface of white cells [177]. Again, these activities contribute to the resolution of inflammation under normal circumstances but how they influence sepsis is unclear. Indeed, clinical trials of a number of “anti-inflammatory” mediators have not demonstrated benefit in patients with sepsis [178].

#### What is not known—gaps in knowledge

• How do cytokines (IL-1RA, IL-4, IL-10, and TGF-β) with known anti-inflammatory effects contribute to the resolution of sepsis? What other white cell mediators contribute?

• Does the activity of IL-1RA, IL-4, IL-10, and/or TGF-β contribute to sepsis-associated immunosuppression? What other mediators are involved?

• Is the balance between pro- and anti-inflammatory activities that mediate recovery from “normal” inflammation lost in the resolution of sepsis?

2. What processes in immune cells other than cytokine elaboration and release contribute to the resolution of sepsis?

#### What is known

As mentioned, sepsis is associated with a poorly understood defect in bacterial engulfment and killing by leukocytes [27]. In normal inflammation, the endocytosis-mediated elimination of HLA-DR for the surface of white cells by IL-10 plays an important role [177]. Changes in lymphocyte expression of the inhibitory receptor programmed death 1 (PD-1) and changes of T cell receptor diversity occur during healthy recovery from nonseptic conditions. However, it is unclear whether a similar process in sepsis leads to a healthy resolution of inflammation or if it represents an additional manifestation of sepsis pathobiology. PD-1 has been implicated in sepsis-associated immune suppression in both animals and humans [179, 180]. PD-1 overexpression by circulating T cells has been detected in septic patients and correlates with worse outcomes [179]; further, normalization of PD-1 expression at day 7 was noted in septic shock survivors [180].

A number of processes are known to contribute to limitations in normal/balanced inflammation. For example, lipoxin A4 regulates MCP-1 and nonphlogistic monocyte recruitment and stops LTB4-stimulated PMN influx [181]. Similarly, maresins, protectins, and resolvins limit further PMN influx to the site and stimulate efferocytosis and the clearance of cellular debris by resolving macrophages [181]. Autophagy-related proteins that act as critical regulators of caspase-1 activation (e.g., beclin-1 and LC3B) in vitro and in vivo contribute to normal/balanced inflammation by preventing accumulation of physiologically abnormal mitochondria [182]. miRNAs, such as programmed miR-466l expression, are temporally and differentially expressed during the resolution of balanced inflammation [183]. Impairment of any of these processes might contribute to failed resolution of sepsis.

#### What is not known—gaps in our understanding—directions for future research

• Sepsis is associated with impairment in a number of processes that contribute to resolution of normal/balanced inflammation. Does resolution of this defect contribute to recovery from sepsis? Or, conversely, does recovery from sepsis lead to resolution of these abnormalities?

• Does the PD-1 pathway exacerbate sepsis pathobiology or does it enhance recovery?

• Do lipid mediators, autophagy, or miRNA, which contribute to resolution of balanced inflammation, also contribute to recovery from sepsis?

• Resolution of inflammation in states other than sepsis involves specific intracellular pathways and/or events in immune cells. Which of these events/intracellular pathways also contribute to resolution of sepsis? What pathways/events not involved in other states are important for resolution of inflammation in sepsis?

• What events in immune cells that contribute to resolution of nonseptic inflammation promote sepsis-induced immunosuppression, thereby delaying the resolution of sepsis?

B) What processes related to metabolism and/or bioenergetics contribute to recovery from cellular and subcellular dysfunction?

#### What is known

The catabolic state produced by sepsis causes alterations in protein breakdown and is characterized by a reduction in body weight, lean body mass, skeletal muscle mass, and fat mass. Metabolism, in general, is impaired, but compensatory mechanisms such as endocrine activity and enhanced substrate availability mask these abnormalities. For example, while global hepatic glucose production is higher than that observed under nonseptic conditions, this increase reflects high levels of catecholamines, corticosteroids, and glucagon. The response of the liver in the absence of sepsis to equivalent levels of hormonal stimulation far exceeds that observed during sepsis [184]. Hepatic inefficiency at least in part reflects impairment of intracellular signal transduction pathways for hormones and other mediators [12, 21, 24, 185, 186]. Although it is likely that the recovery mechanisms that restore the balance between catabolism and anabolism differ from those that initiated the imbalance, the actual process is poorly described. Importantly, lean body mass may be depleted in sepsis but cell death is not a significant feature in most organs and functional recovery does not appear to be limited by the regenerative capacity of the tissue, perhaps because solid organ mass does not appear to be affected [187]. It is essential to note that metabolic changes during sepsis recovery may be linked to other subcellular mechanisms, including autophagy, apoptosis, and proteasome activity [182, 188–190].

Some aspects of metabolic recovery have been examined in experimental models, most often using the CLP model in rodents. Crowell et al. [190] studied the regulation of skeletal muscle protein balance during recovery from CLP in mice. These investigators noted (1) persistence of CLP-induced proteolysis persisted during the recovery phase, (2) a period of enhanced muscle protein synthesis that was mediated by activation of Akt-TSC2-mTORC1 signal transduction, and (3) apparent delay of complete restoration of muscle mass that was in part explained by continued stimulation of the ubiquitin-proteasome pathway-mediated proteolysis.

Although the erosion of lean body mass and skeletal muscle loss have been studied [190, 191], examination of sepsis-induced changes in fat mass and adipocyte biology has been less thorough. The same group also showed that white adipose tissue stimulated continued inflammation with activation of the inflammasome, the ubiquitin-proteasome pathway, and autophagy, even when the infectious source was excised. These changes overlapped with alterations in skeletal muscle. However, release of uncoupling protein 1 during recovery, suggesting a “browning” of this white adipose tissue, was associated with diminution of inflammasome activation and autophagy as well as limited lipolysis and lipogenesis [192]. Again, these findings suggest that sepsis-induced changes in substrate metabolism persist into the recovery phase but that distinct recovery mechanisms exist.

Some of the mechanisms that modulate the balance between protein breakdown and synthesis also contribute to control of autophagy, a process that facilitates the turnover of organelles and intracellular protein. Both skeletal muscle turnover and autophagy are tightly controlled by mTORC1 and adenosine monophosphate-activated protein kinase (AMPK) [193]. Recovery of AMPK activity following CLP was associated with reduced severity and less profound lung injury [194] as well as improved bacterial clearance [195].

As described under question 2, bioenergetics are also significantly altered by sepsis. Mitochondrial dysfunction has been demonstrated in human sepsis and in animal models, including CLP [33, 196–200]. Fink termed this block in oxidative phosphorylation “cytopathic hypoxia” [201]. In 2008, Carré and Singer [202] suggested a link between recovery from sepsis and mitochondrial biogenesis. This contention is supported by animal studies demonstrating that recovery was associated with a progressive increase in cytochrome and mitochondrial DNA, followed by recovery of oxygen consumption and resting energy expenditure [203]. Additional investigations using CLP demonstrated improved organ function with reversal or bypass of dysfunction in specific elements of the ETC [204–209]. Recovery in patients was associated with early production of new mitochondria (biogenesis) [210]. Glycolysis contributes substantially to ATP generation in the presence of sepsis-induced mitochondrial dysfunction [39, 45, 48, 56, 57, 198, 211–213]. However, the glycolytic process is complex. Studies in animals have suggested that it is highly dependent on substrate delivery, Na-K transport [214], stimulation by catecholamines [215], and the Warburg effect [49, 216–218].

#### What is not known—gaps in our understanding—directions for future research

1. Does reversal of sepsis-induced changes in metabolism/energetics promote recovery? Or is reversal of sepsis-induced changes simply an indicator that recovery is occurring/has occurred?
2. What pathways both mediate sepsis-induced changes in metabolism and also effect recovery from sepsis? What pathways that modulate sepsis-induced changes in metabolism do NOT mediate recovery, and vice versa? Can any of these pathways be manipulated to initiate or enhance recovery from sepsis?
3. Do unexplored or undiscovered pathways mediate both the development of sepsis-induced changes in metabolism and the recovery from these changes?
4. Can the pathways that mediate recovery from sepsis-induced changes in both protein and fat metabolism be manipulated to initiate or enhance recovery from sepsis?
5. Can the “browning” of fat be enhanced? Can manipulation of “browning” be used to enhance recovery from sepsis?
6. Is the restoration of mitochondrial function necessary for recovery from sepsis?
7. Do interventions that improve the function of individual aspects of mitochondrial function promote recovery from sepsis? Do interventions that have been found to be successful following CLP also enhance recovery from human sepsis?
8. Recovery requires ATP utilization above basal levels. In addition to serving as a source of energy production during sepsis, does ATP production from lactate, that is, glycolysis, meaningfully contribute to recovery from sepsis? What mechanisms regulate sepsis-induced lactate production? Can these mechanisms be manipulated for therapeutic benefit?

C) What endocrine and neuronal pathways contribute to recovery from sepsis?

#### What is known

Endocrine dysfunction is a key characteristic of sepsis; sepsis-induced abnormalities have been reported in the intracellular pathways mediating tissue responses to nearly every hormone [19, 20]. Dysfunction takes two forms. In the acute phase, tissues become hypo-responsive (“peripheral resistance”) and central mechanisms to increase hormone release are activated [19]. Ultimately, however, central secretion of hormones, for example, from the pituitary, decreases (“central suppression”). Restoration of both tissue responses and central elaboration/release have been described in recovery [19]. However, resolution of sepsis-induced defects in endocrine activity may simply be a result of recovery as opposed to a contributing factor.

Neuronal abnormalities are believed to contribute to sepsis pathobiology. For example, recent studies have demonstrated a role for dysfunction in the orexinergic pathway of the hypothalamus [27] and in the basal forebrain cholinergic system [28, 219]. Seminal work by Pavlov and Tracey [220] defined the “cholinergic anti-inflammatory pathway,” which downregulates inflammation in sepsis via activation of vagus nerve efferents, leading to sequential splenic release of norepinephrine and then acetylcholine which in turn downregulates activation of endothelial cells and promotes a pro-resolving phenotype in macrophages and monocytes. Importantly, the vagus-mediated anti-inflammatory pathway is impaired following CLP [220]. Stimulation of the vagus can reverse some elements of the CLP-induced inhibition of this reflex [221].

Cognitive defects that may be analogous to those present in patients with sepsis have been identified following CLP in rodents. These abnormalities have been attributed to a number of underlying causes, including neuroinflammation, a number of different brain regions, most notably the hippocampus and basal forebrain, and in some cases have been amenable to treatment [28, 168, 222–231]. However, the relevance of these abnormalities to human sepsis is unknown.

#### What is not known—gaps in our understanding—directions for future research

1. Is reversal of sepsis-induced endocrine/neuronal defects a cause or an effect of recovery?
2. What specific hormones and/or neuronal pathways contribute to recovery? Are there pathways that are not operative in the pathogenesis of sepsis that are involved in recovery?
3. Do strategies that counteract or reverse endocrine or neuronal dysfunction contribute to recovery?
4. What additional neuronal pathways contribute to the pathogenesis of sepsis?
5. What neuronal abnormalities, pathways, and defects contribute to sepsis-induced short-term neurobehavioral abnormalities and long-term cognitive dysfunction?

## Summary

This document expands upon topics in “Basic Science Research” that were specifically identified as priorities by the SSC Research Committee [2, 3]. The goal of the authors and the committee was to provide the critical care community with a detailed, well-balanced, and highly informative summary regarding topics specifically identified by the members of the SSC Research Committee as a whole. Our focus, and the focus of future publications, is on “what is known,” followed by enumeration of ongoing research priorities and questions that we believed are unanswered. Each of the basic research areas that we have focused on has high translational potential. We have called upon our collective insights as critical care practitioners and as investigators focusing on basic science research in sepsis. We have also included recommendations from other members of the SSC Research Committee that reflect long-term involvement in the critical care community as a whole.

We recognize that members of the critical care community have diverse backgrounds and interests and that many are more clinically focused. We therefore have attempted to provide a balanced and accessible review and perspective and to provide material that is useful to all involved in our specialty. Importantly, the process used to generate the topics discussed here is, by its nature, subjective. Members of the committee all have interests in/commitments to the study of specific topics. We have tried to limit the effects of personal interest/bias on the choice of topics. Similarly, some areas of interest to some readers, of necessity, will not be represented in this document. Undoubtably some seeming omissions will be addressed in future papers.

We are grateful to the leadership of the SSC, the ESICM, and the SCCM for providing the opportunity and for their continued commitment to critical care research.

## Appendix

Members of the Surviving Sepsis Campaign Research Committee: Craig M. Coopersmith, MD, MCCM, Co-chair (Atlanta, GA); Daniel De Backer, MD, Co-chair (Brussels, Belgium); Massimo Antonelli, MD (Rome, Italy); Clifford S. Deutschman, MS, MD, MCCM (Manhasset, NY); Laura Evans, MD, MSc, FCCM (Seattle, WA—Consultant); Ricard Ferrer-Roca, MD, PhD (Barcelona, Spain); Judith Hellman, MD (San Francisco, CA); Sameer Jog, MD; Jozef Kesecioglu, MD, PhD (Utrecht, The Netherlands); Ishaq Lat, PharmD (Chicago, IL); Mitchell M. Levy, MD, MCCM (Providence, RI); Flavia Machado, MD, PhD (Sao Paulo, Brazil); Greg Martin, MD, MSc, FCCM (Atlanta, GA); Ignacio Martin-Loeches, MD, PhD, FJFICMI; Mark E. Nunnally, MD, FCCM (New York, NY); and Andrew Rhodes, MBChB, FRCP, FRCA, FFICM (London, United Kingdom—Consultant).
